# Physiotherapists’ knowledge, attitude and practice of motor learning in stroke rehabilitation

**DOI:** 10.4102/sajp.v82i1.2350

**Published:** 2026-06-29

**Authors:** Elmi Meyer, Helena W. Nel, Dineo Mahlangu, Riette Nel

**Affiliations:** 1 Department of Physiotherapy, Faculty of Health Sciences, University of the Free State, Bloemfontein, South Africa; 2 Department of Biostatistics, Faculty of Health Sciences, University of the Free State, Bloemfontein, South Africa

**Keywords:** knowledge, attitude, practice, motor impairment, motor learning, survey

## Abstract

**Background:**

To improve motor impairment in stroke survivors, physiotherapists are encouraged to structure exercise sessions in such a way that motor learning is optimised in stroke survivors. No studies have described the knowledge, attitudes or practices of South African physiotherapists regarding motor learning in stroke rehabilitation.

**Objectives:**

To describe the knowledge, attitudes and practices of South African physiotherapists regarding motor learning in stroke rehabilitation.

**Method:**

Convenient and snowball sampling techniques were used. Self-compiled questionnaires were sent to members of the South African Society of Physiotherapy (SASP) via an electronic survey platform.

**Results:**

Forty physiotherapists completed the survey. Respondents’ main area of interest was spread widely: the most indicated area was neuromusculoskeletal physiotherapy (*n* = 16, 40%), followed by adult neurological rehabilitation (*n* = 7, 17.5%). Fifty per cent of respondents treated between 1 stroke survivor and 10 stroke survivors with motor impairment over the past year. Most respondents (*n* = 36, 90%) had moderate knowledge of motor learning in stroke rehabilitation. However, study findings indicate knowledge gaps in several areas. Most respondents (*n* = 37, 92.5%) considered the application of motor learning principles to be an important tool to improve motor impairment in stroke survivors. Respondents inconsistently applied motor learning recommendations from the literature into stroke rehabilitation.

**Conclusion:**

The study revealed knowledge and practice gaps in several motor learning areas and inconsistent application of motor learning recommendations from the literature into stroke rehabilitation.

**Clinical implications:**

Findings highlight the need for further post-graduate training to allow physiotherapists to confidently apply motor learning principles in stroke rehabilitation.

## Introduction

The physiotherapy approach to the treatment of stroke survivors has evolved over time (Pollock et al. 2014).

In the 1950s, various approaches to the treatment of stroke survivors were developed based on the neurophysiology knowledge available at the time, for example, the method of Bobath (1990). Since the 1980s, the focus of physiotherapy in stroke rehabilitation has gradually shifted from an approach based mainly on neurophysiology principles to a more eclectic approach that incorporates principles of motor learning and biomechanics (Kleynen et al. 2020).

For this study, motor learning was defined as a set of internal processes, involving practise and exercise, resulting in the acquisition of motor skills and leading to a relatively permanent improvement in skilled motor behaviour (Kwakkel et al. 2023; Riga et al. 2022; Schmidt & Lee 2011). In lay man’s terms, motor learning is the process, involving practice and exercise, by which a person learns to do a new movement smoother, faster and more accurately (Riga et al. 2022; Skurvydas et al. 2020). According to Bramley et al. (2018:366), physiotherapists should structure exercise sessions with stroke survivors according to motor learning principles, defined as ‘key principles to optimise the practise and retention of motor skills’. In the multi-disciplinary stroke rehabilitation team, physiotherapists often focus their attention on improving motor impairment in stroke survivors (Langhorne, Coupar & Pollock 2009). Motor impairment is defined as ‘a loss or limitation of function in muscle control or movement or a limitation in mobility’ (Langhorne et al. 2009:741). A systematic review by Veerbeek et al. (2014) found that repetition and the application of motor learning principles are the driving factors behind most physiotherapy interventions that improve motor impairment in stroke survivors. More recently, results from the Interdisciplinary Comprehensive Arm Rehabilitation Evaluation (ICARE) trial suggested that exercise based on motor learning principles may increase the rate at which motor impairments recover in stroke survivors (Lewthwaite et al. 2018).

There is consensus among researchers that the way in which physiotherapist structure exercise sessions with stroke survivors influences motor learning in these patients (Levac et al. 2011, 2016; Winstein et al. 2014). The *Koninklijk Nederlands Genootschap voor Fysiotherapie* [Royal Dutch Society for Physiotherapy (KNGF)] Clinical Practice Guideline (2014) summarises the exercise variables that determine the efficacy of motor learning in stroke survivors: Intensity of exercise, difficulty of exercise, feedback from the physiotherapist, variability of exercise, specificity of exercise and the motivation and active engagement of the stroke survivor. Kleim and Jones (2008) translated motor learning knowledge gained from animal studies into guidelines for structuring human stroke rehabilitation. Levin and Demers (2021); Maier, Ballester and Verschure (2019); Winstein and Kay (2015) and Muratori et al. (2013) also provide recommendations for physiotherapists to structure exercise sessions for optimal motor learning in stroke survivors. Kleynen et al. (2020) describe a framework developed to help physiotherapists apply motor learning in stroke rehabilitation.

Stroke is a leading cause of disability in South Africa (Taylor & Ntusi 2019). The South African Contextualized Stroke Rehabilitation Guideline (SA-cSRG 2019) is the only Clinical Practice Guideline that provides evidence-based guidance for stroke rehabilitation in South Africa. Though motor learning is not mentioned directly, the SA-cSRG contains a recommendation for the rehabilitation of stroke survivors, which includes all the key exercise variables known to promote motor learning in stroke survivors. This recommendation reads: ‘Patients should engage in training that is meaningful, engaging, progressively adaptive, intensive, task-specific and goal oriented in an effort to improve transfer skills and mobility’ (SA-cSRG 2019:81).

Though various studies in high-income countries investigated motor learning in stroke rehabilitation in recent years (Almarwania & Aldawsary 2023; Janssen et al. 2020; Johnson et al. 2023), no previous studies have investigated motor learning in the context of South African stroke rehabilitation. Gaining an understanding of what South African physiotherapists know about motor learning in the context of stroke rehabilitation, how they feel about motor learning in stroke rehabilitation and how they apply motor learning principles in stroke rehabilitation is a first step towards ensuring South African physiotherapists’ optimal use of motor learning in stroke rehabilitation, which may ultimately lead to improved recovery of motor impairment in stroke survivors. Thus, this study aimed to describe the knowledge, attitudes and practices of South African physiotherapists regarding motor learning in stroke rehabilitation.

## Research methods and design

### Study design

The study used a descriptive survey to assess the knowledge, attitudes and practices of South African physiotherapists. The data collection instrument consisted of a self-compiled questionnaire. EvaSys was used as the online survey platform. A convenient sampling strategy was used: the South African Society of Physiotherapy (SASP) distributed the survey electronically (via bulk email) to its members. Snowball sampling was also used, as physiotherapists reached through the SASP could share the survey with other physiotherapists who were not members of the SASP.

Physiotherapists registered with the Health Professions Council of South Africa (HPCSA) were included in the study – this included community service physiotherapists and independent physiotherapy practitioners.

Participation in the study was voluntary; respondents provided electronic consent to participate in the study.

Participation in the study was confidential. Data were anonymised before data analysis.

### Sample size calculation

There were 8749 physiotherapists registered with the HPCSA in South Africa in April 2023 (HPCSA 2023).

The sample size was calculated using a confidence level of 95%, a margin of error of 5%, a population proportion of 50% and a population size of 8749. The ideal sample size for the study was 369 respondents.

### Data collection instrument

The data collection instrument was a self-compiled questionnaire, which took approximately between 15 min to 20 min to complete. The questionnaire was developed according to Knowledge Attitude and Practice (KAP) guidelines (Andrade et al. 2020; Kaliyaperumal 2004; Launiala 2009) and based on the literature regarding motor learning. To enhance the face and content validity of the questionnaire, two experts in the field of neurorehabilitation reviewed the draft questionnaire in terms of content, the correct use of terminology and the extent to which data obtained through the questionnaire would answer the study objectives. The questionnaire was piloted on two consenting physiotherapists.

The questionnaire consisted of five sections. Section one (Informed consent) explained the aim of the study.

At the end of Section one, physiotherapists ticked a box to provide electronic consent to participate in the study. Only respondents who provided consent were able to continue to Section two of the questionnaire.

Section two (Demographic information) consisted of questions used to elicit respondent characteristics and the characteristics of the sample group

Section three (Knowledge) consisted of 10 questions to which respondents had to answer true, false or unsure.

Respondents were awarded one point for every question they answered correctly, while zero points were awarded for incorrect answers or questions to which respondents answered ‘unsure’. Respondents’ knowledge was classified as poor if they answered three or less of the ten questions correctly, moderate if they answered four to seven of the ten questions correctly and good if they answered eight or more of the ten questions correctly.

Section four (Attitude) determined respondents’ attitudes towards motor learning in stroke rehabilitation.

Respondents were asked to indicate their level of agreement with five given statements: (1) strongly agree (2) agree (3) unsure (4) disagree, and (5) strongly disagree). Respondents’ attitudes were not classified as positive or negative.

Instead, respondents’ attitudes towards various aspects of motor learning in stroke rehabilitation were determined by analysing respondents’ responses on a question-by-question basis. Section five (Practice) consisted of questions to determine respondents’ self-reported implementation of motor learning principles during stroke rehabilitation. Respondents’ responses were analysed on a question-by-question basis; respondents’ practices were not classified as effective or ineffective.

### Data collection and analysis

The survey was conducted for 10 weeks (03 June 2024–12 August 2024). According to their policy for electronic research surveys, the SASP only sent the survey once (via bulk email) to their members. Physiotherapists reached through the SASP could share the survey with other physiotherapists who were not members of the SASP. The link to the survey was also posted on the SASP’s website and Facebook page for the duration of the data collection period.

After the data collection period ended, the data were exported for all completed questionnaires from EvaSys into a Microsoft Excel spreadsheet. This Microsoft Excel spreadsheet was password protected to allow only the researcher access to the data, and the data were saved on Figshare. By comparing respondents’ HPCSA numbers, the researcher made sure the same physiotherapist did not complete the questionnaire more than once. A second Microsoft Excel spreadsheet was created, and data were de-identified by deleting respondents’ HPCSA numbers and allocating a number to every research participant. The researcher shared the Microsoft Excel spreadsheet with de-identified data via Figshare for data analysis. Data analysis was done by the Department of Biostatistics at the University of the Free State with the assistance of the biostatistician.

Descriptive statistics (frequencies and percentages) were calculated for categorical data. The distribution of the numerical data was skew; therefore, medians and interquartile ranges were calculated as summary statistics. Data were analysed using Statistical Analysis Software (SAS/STAT) software, Version 9.4 of the SAS system for Windows, Copyright^©^ 2016 SAS Institute Inc. SAS and all other SAS Institute Inc. products or service names are registered trademarks of SAS Institute Inc., Cary, North Carolina, United States (US).

### Ethical considerations

Ethical clearance to conduct this study was obtained from the Health Sciences Research Ethics Committee of the University of the Free State (No. UFS-HSD2023/2670/2603). Permission was also obtained from the Presidential Executive Committee of the SASP for the survey to be distributed via the SASP’s bulk emailing system and to be uploaded on the SASP website and Facebook page.

## Results

### Respondent characteristics

Forty physiotherapists completed the survey – 10.8% of the ideal sample size. Most respondents (*n* = 33, 82.5%) were practicing in the private health care sector. Respondents’ main area of interest was spread widely, the most indicated area was: (1) neuromusculoskeletal physiotherapy (*n* = 16, 40%), followed by, (2) adult neurological rehabilitation (*n* = 7, 17.5%). While most respondents (*n* = 39, 97.5%) treated stroke survivors with motor impairment over the last year, half of the respondents (*n* = 20, 50%) only treated between 1 stroke survivor and 10 stroke survivors with motor impairment over the last year (Table 1).

**TABLE 1:** Demographic characteristics of respondents (*N* = 40).

Variable	*n*	%
**Job description**		
Community service physiotherapist	0	0
Independent physiotherapy practitioner	40	100
**Practicing as a physiotherapist (years)**		
1–5	11	27.5
6–10	11	27.5
11–20	11	27.5
> 20	7	17.5
**Work environment**		
Public sector	7	17.5
Private sector	33	82.5
**Gender**		
Male	3	7.5
Female	37	92.5
**Highest qualification in physiotherapy**		
BSc Physiotherapy	36	90.0
MSc Physiotherapy	3	7.5
Other	1	2.5
**Main area of interest in physiotherapy**		
Cardiopulmonary rehabilitation	6	15.0
Adult neurological rehabilitation	7	17.5
Paediatric neurological rehabilitation	1	2.5
Neuromusculoskeletal physiotherapy	16	40.0
Geriatrics	1	2.5
Pain management	3	7.5
Gender health	1	2.5
Sport physiotherapy	5	12.5
**Number of stroke survivors with motor impairment treated over the past year**		
None	1	2.5
1–10	20	50.0
11–20	9	22.50
21–30	3	7.5
> 30	7	17.5

*Source:* Adapted for this study from Meyer, E., 2024, ‘Knowledge, attitude and practice of South African physiotherapists regarding motor learning in adult stroke rehabilitation’, Masters thesis, Department of Physiotherapy, University of the Free State

BSc, Bachelor of Science; MSc, Masters of Science.

### Knowledge of motor learning in stroke rehabilitation

Most respondents (*n* = 36, 90%) had moderate knowledge of motor learning in the context of stroke rehabilitation. Four respondents (10%) had poor knowledge. The median score for respondent knowledge was 5.0 with an interquartile range of 4.0–5.5. None of the respondents showed good knowledge of motor learning in stroke rehabilitation (Table 2).

**TABLE 2:** Respondents’ knowledge of motor learning in stroke rehabilitation (*N* = 40).

Statement	Correct answers		Incorrect answers		Unsure	
	*n*	%	*n*	%	*n*	%
Motor impairment is not a common impairment observed in stroke survivors.	29	72.5	4	10.0	7	17.5
Modern motor control approaches are based on the reflex and hierarchic models of motor control.	7	17.5	14	35.0	19	47.5
Recovery in stroke survivors occurs through a combination of spontaneous and learning-dependent processes.	38	95.0	2	5.0	0	0.0
The first 6 months – 12 months after a stroke is known as the window of opportunity where interventions targeting brain repair and recovery should be utilised to optimise the extent of recovery in stroke survivors.	0	0	38	95.0	2	5.0
In adult stroke survivors, motor learning is associated with neuroplastic changes in spared sections of the brain (*n* = 39)	37	95.0	2	5.0	0	0
Motor learning occurs in stages	32	80.0	3	7.5	5	12.50
Motor learning is facilitated when stroke survivors actively engage in problem-solving while training motor tasks.	36	90.0	2	5.0	2	5.0
If a stroke survivor’s performance of a task improves from the beginning to the end of a physiotherapy session, it can be assumed that motor learning has occurred.	4	10.0	33	82.5	3	7.5
Several standardised outcome measures are available to measure or determine motor learning.	4	10.0	20	50.0	16	40.0
Any training that involves the affected limb will lead to motor learning and neuroplastic changes in the brains of stroke survivors.	6	15.0	23	57.5	11	27.50

*Source:* Adapted for this study from Meyer, E., 2024, ‘Knowledge, attitude and practice of South African physiotherapists regarding motor learning in adult stroke rehabilitation’, Masters thesis, Department of Physiotherapy, University of the Free State

Most respondents knew that motor impairment is a common impairment in stroke survivors (*n* = 29, 72.5%), that motor learning occurs in stages (*n* = 32, 80%) and that motor learning is facilitated when stroke survivors actively engage in problem-solving while practising movements (*n* = 36, 90%). Most respondents (*n* = 37, 95%) also knew that, in stroke survivors, motor learning is associated with neuroplastic changes in spared sections of the brain. Gaps in respondents’ knowledge were found in several areas: modern motor control approaches, motor learning outcome measures, the distinction between learning and performance in motor learning and the type of exercise that promotes motor learning in stroke survivors. In addition, no respondent answered the question correctly about the optimal time frame after stroke when physiotherapy interventions should be delivered to maximise motor impairment in stroke survivors (Table 2).

### Attitude towards motor learning in stroke rehabilitation

Most respondents (*n* = 37, 92.5%) considered the application of motor learning principles to be an important tool to improve motor impairment in stroke survivors. All respondents believed that an important aim of physiotherapy in stroke rehabilitation is to help stroke survivors re-learn the everyday motor skills they need to become more independent, and that physiotherapists should regard stroke survivors as active participants and learners instead of passive recipients of therapy. While most respondents (*n* = 33, 84.6%) felt the way in which physiotherapists structure their physiotherapy sessions can influence motor learning in stroke survivors, the remaining six respondents (15.38%) were unsure (Table 3).

**TABLE 3:** Respondents’ attitude towards motor learning in stroke rehabilitation (*N* = 40).

Statement	Strongly agree		Agree		Unsure		Disagree		Strongly disagree	
	*n*	%	*n*	%	*n*	%	*n*	%	*n*	%
The application of motor learning principles is a valuable tool for physiotherapists to improve motor impairment in stroke survivors.	22	55.0	15	37.5	3	7.5	0	0	0	0
An important aim of physiotherapy in stroke rehabilitation is to help stroke survivors re-learn the everyday motor skills they need to become more independent.	33	82.5	7	17.5	0	0.0	0	0	0	0
The way in which physiotherapists structure their physiotherapy sessions can influence motor learning in stroke survivors (*n* = 39)	21	54.0	12	31.0	6	16.0	0	0	0	0
Physiotherapists should regard stroke survivors as active participants and learners instead of passive recipients of therapy.	35	87.5	5	12.5	0	0.0	0	0	0	0
A stroke survivor’s level of motivation to engage in physical rehabilitation influences motor learning.	30	75.0	10	25.0	0	0.0	0	0	0	0

*Source:* Adapted for this study from Meyer, E., 2024, ‘Knowledge, attitude and practice of South African physiotherapists regarding motor learning in adult stroke rehabilitation’, Masters thesis, Department of Physiotherapy, University of the Free State

### Motor learning practice

Most respondents (*n* = 25, 64.1%) reported exercising with stroke survivors for 30 min – 45 min per day, between 1 day and 4 days a week. Most respondents (*n* = 26, 66.67%) let a stroke survivor exercise at a level of difficulty that was well within the stroke survivor’s physical abilities. Variability of practice was used by most respondents (*n* = 28, 70%) in exercise sessions with stroke survivors. Most respondents (*n* = 37, 92.5%) used feedback with an internal focus of attention to give feedback to stroke survivors during exercise sessions, and most respondents (*n* = 25, 62.5%) provided less frequent feedback as the stroke survivor became more skilled in a movement. In exercise sessions with stroke survivors aimed at addressing motor impairment, 22 respondents (55%) often included task-specific training. When practising functional tasks with a stroke survivor, the minority of respondents (*n* = 7, 32.2%) always or often set-up the treatment environment to reflect the stroke survivor’s familiar home and/or community environment (Table 4).

**TABLE 4:** Respondents’ self-reported implementation of motor learning in stroke rehabilitation (*N* = 40).

Variable	*n*	%
**In the clinical setting where you work, for how many minutes per day would a stroke survivor typically exercise with a physiotherapist?**		
0 min – 30 min	7	17.5
31 min – 45 min	13	32.5
46 min – 60 min	11	27.5
61 min – 120 min	4	10.0
We do not treat adult stroke survivors in the clinical setting where I work.	5	12.5
**In the clinical setting where you work, how many days of the week would a stroke survivor typically receive physiotherapy exercise sessions?**		
1 day – 4 days	13	32.5
5 days	2	5.0
6 days	10	25.0
7 days	9	22.5
We do not treat adult stroke survivors in the clinical setting where I work.	6	15.0
**When training with stroke survivors, do you structure your physiotherapy session to present them with a motor or environmental problem for which the solution requires an appropriately planned and executed movement?**		
Always	8	20.0
Often	16	40.0
Sometimes	11	27.5
Seldom	2	5.0
Never	3	7.5
**In your physiotherapy session, a stroke survivor is practising a sit to stand task. With motor learning in mind, how difficult would you make the exercise? (*n* = 39)**		
Well within the stroke survivor’s current ability.	26	66.67
Just manageable for the stroke survivor (at the edge of the stroke survivor’s current ability).	13	33.33
**A stroke survivor is practising a sit to stand task. With motor learning in mind, would you** …		
… provide feedback with an external focus.	3	7.5
… provide feedback with an internal focus.	37	92.5
**In your physiotherapy session, a stroke survivor is practising transfers from a wheelchair to another surface. With motor learning in mind, would you …**		
… let the stroke survivor practise transfers from a wheelchair to a hard chair, a wheelchair to a bed, a wheelchair to a toilet and a wheelchair to a car?	28	70.0
… let the stroke survivor practise to transfer between a wheelchair and a plinth in the rehabilitation gym?	12	30.0
**A stroke survivor has learned the basic skill of opening and closing her water bottle. With motor learning in mind, how would you use feedback (verbal or nonverbal) to guide him as he refines the skill?**		
I would give more frequent feedback than I did when he was learning the basic skill.	8	20.0
I would give the same amount of feedback than I did when he was learning the basic skill.	7	17.5
I would give less frequent feedback than I did when he was learning the basic skill.	25	62.5
**With stroke survivors who are strong enough to engage in task-specific training, how often do you include task-specific training in your treatment sessions aimed at improving motor impairment?**		
Always	8	20.0
Often	22	55.0
Sometimes	7	17.5
Seldom	2	5.0
Never	1	2.5
**When practising functional tasks with survivors, do you set up the treatment environment to reflect their familiar home and/or community environment?**		
Always	5	12.5
Often	8	20.0
Sometimes	12	30.0
Seldom	7	17.5
Never	8	20.0

*Source:* Adapted for this study from Meyer, E., 2024, ‘Knowledge, attitude and practice of South African physiotherapists regarding motor learning in adult stroke rehabilitation’, Masters thesis, Department of Physiotherapy, University of the Free State

### Knowledge, attitude and practice of respondents who regularly treated stroke survivors

Seven respondents treated more than 30 stroke survivors with motor impairment over the past year. The data gained from these respondents were analysed along with the data from the rest of the respondents. However, the knowledge, attitude and practice of the respondents who treated stroke survivors on a regular basis were also investigated more closely.

Six respondents had moderate knowledge of motor learning in the context of stroke rehabilitation, while one respondent had poor knowledge. One respondent answered three knowledge questions of the 10 knowledge questions correctly, another respondent answered 4 questions correctly, four respondents answered 5 questions of the 10 questions correctly and one respondent answered 6 questions of the 10 questions correctly.

All seven respondents considered the application of motor learning principles to be an important tool to improve motor impairment in stroke survivors. Respondents all believed that an important aim of physiotherapy in stroke rehabilitation is to help stroke survivors re-learn the everyday motor skills they need to become more independent, and that physiotherapists should regard stroke survivors as active participants and learners instead of passive recipients of therapy. Every respondent felt the way in which physiotherapists structure their physiotherapy sessions can influence motor learning in stroke survivors.

The amount of time respondents spent exercising with stroke survivors varied widely. Most respondents (*n* = 6, 85.7%) let a stroke survivor exercise at a level of difficulty that was well within the stroke survivor’s physical abilities. Most respondents (*n* = 5, 71.4%) used verbal feedback with an internal focus of attention to guide a stroke survivor during exercise sessions and provided less frequent feedback (*n* = 5, 71.4%) as the stroke survivor became more skilled in a movement. Respondents often (*n* = 5, 71.4%) or always (*n* = 2, 28.6%) included task-specific training in exercise sessions aimed at improving motor impairment in stroke survivors. Four respondents of the seven respondents seldom (*n* = 3, 42.9%) or never (*n* = 1, 14.3%) let stroke survivors practice functional tasks in a treatment area set up to reflect the stroke survivors’ home or community environment (Table 5). In terms of motor learning practices, respondents followed some best-practice recommendations from the literature, but not all.

**TABLE 5:** Motor learning practices of respondents who regularly treated stroke survivors (*n* = 7).

Variable	*n*	%
**How much time do you spend exercising with stroke survivors in a week?**		
0 min – 30 min, 7 days a week	1	14.3
31 min – 45 min, 1 day – 4 days a week	1	14.3
31 min – 45 min, 7 days a week	1	14.3
46 min – 60 min, 5 days a week	1	14.3
46 min – 60 min, 6 days a week	1	14.3
61 min – 120 min, 6 days a week	2	28.6
**When training with stroke survivors, do you structure your physiotherapy session to present them with a motor or environmental problem for which the solution requires an appropriately planned and executed movement?**		
Always	2	28.6
Often	3	42.9
Sometimes	2	28.6
Seldom	0	0
Never	0	0
**In your physiotherapy session, a stroke survivor is practising a sit to stand task. With motor learning in mind, how difficult would you make the exercise?**		
Well within the stroke survivor’s current ability.	6	85.7
Just manageable for the stroke survivor (at the edge of the stroke survivor’s current ability).	1	14.3
**A stroke survivor is practising a sit to stand task. With motor learning in mind, would you** …		
… provide feedback with an external focus.	2	28.6
… provide feedback with an internal focus.	5	71.4
**In your physiotherapy session, a stroke survivor is practising transfers from a wheelchair to another surface. With motor learning in mind, would you … (*n* = 40)**		
… let the stroke survivor practise transfers from a wheelchair to a hard chair, a wheelchair to a bed, a wheelchair to a toilet and a wheelchair to a car?	5	71.4
… let the stroke survivor practise to transfer between a wheelchair and a plinth in the rehabilitation gym?	2	28.6
**A stroke survivor has learned the basic skill of opening and closing her water bottle. With motor learning in mind, how would you use feedback (verbal or nonverbal) to guide him as he refines the skill?**		
I would give more frequent feedback than I did when he was learning the basic skill.	1	14.3
I would give the same amount of feedback than I did when he was learning the basic skill.	1	14.3
I would give less frequent feedback than I did when he was learning the basic skill.	5	71.4
**With stroke survivors who are strong enough to engage in task-specific training, how often do you include task-specific training in your treatment sessions aimed at improving motor impairment?**		
Always	2	28.6
Often	5	71.4
Sometimes	0	0
Seldom	0	0
Never	0	0
**When practising functional tasks with survivors, do you set up the treatment environment to reflect their familiar home and/or community environment? (*n* = 7)**		
Always	1	14.3
Often	1	14.3
Sometimes	1	14.3
Seldom	3	42.9
Never	1	14.3

*Source:* Adapted for this study from Meyer, E., 2024, ‘Knowledge, attitude and practice of South African physiotherapists regarding motor learning in adult stroke rehabilitation’, Masters thesis, Department of Physiotherapy, University of the Free State

## Discussion

The study aimed to describe the knowledge, attitudes and practices of South African physiotherapists regarding motor learning in stroke rehabilitation. Forty respondents completed the survey. Respondents’ main area of interest in physiotherapy was spread widely; adult neurological rehabilitation was not the main area of interest of most respondents. Eighty per cent of respondents worked in the private health care sector. Fifty per cent of respondents did not treat stroke survivors on a regular basis. Although most respondents had moderate knowledge of motor learning in stroke rehabilitation, study findings indicate knowledge gaps in several areas. More than 90% of respondents considered the application of motor learning principles to be an important tool to improve motor impairment in stroke survivors. Respondents inconsistently applied motor learning recommendations from the literature into stroke rehabilitation.

### Respondent characteristics

When drawing conclusions from the study, it is essential to keep in mind that adult neurological rehabilitation was not the main area of interest of most respondents, and half of the respondents did not regularly treat stroke survivors. If only physiotherapists with a special interest in stroke rehabilitation had participated in the study, the study outcomes likely would have been different.

### Knowledge of motor learning in stroke rehabilitation

Most respondents had moderate knowledge of motor learning in the context of stroke rehabilitation. This aligns with Atun-Einy and Kafri (2019, 2021), who reported that Israeli physiotherapists had only a partial understanding of the core concepts of motor learning in physiotherapy. Although moderate motor learning knowledge may seem adequate, an item-by-item analysis of the knowledge questions revealed gaps in respondents’ knowledge (Table 2), which might affect physiotherapy clinical practice.

Most respondents indicated that the optimal time frame for physiotherapy interventions to maximise motor recovery in stroke survivors is the first 6 months – 12 months after stroke (Table 2). In fact, the optimal time frame is the first 3 months – 6 months post stroke (Bernhardt et al. 2017). Physiotherapists under this misconception may not fully appreciate the need to challenge stroke survivors with motor impairment to exercise intensely during this optimal period. Most respondents indicated that any type of training involving the affected limb will promote motor learning and facilitate neuroplasticity in stroke survivors (Table 2), not knowing that only exercises structured according to motor learning principles will do so (Kleim & Jones 2008; Krakauer 2006). Thus, motor learning in stroke survivors may not be optimal during treatment sessions from physiotherapists who lack knowledge in this area.

### Attitude towards motor learning in stroke rehabilitation

Most respondents considered the application of motor learning principles to be an important tool to improve motor impairment in stroke survivors (Table 3), a belief substantiated by research (Veerbeek et al. 2014). In line with research recognising the importance of stroke survivor moving actively during exercise sessions to improve motor impairment (Dorsch et al. 2023), all respondents believed that physiotherapists should regard stroke survivors as active participants and learners instead of passive recipients of therapy (Table 3).

There is consensus among researchers that the way in which physiotherapists structure exercise sessions with stroke survivors influences motor learning in these patients (Levac et al. 2011, 2016; Winstein et al. 2014). Some respondents, however, were uncertain if the way physiotherapists structure exercise sessions with stroke survivors can influence motor learning in these patients (Table 3), which might suggest unfamiliarity with motor learning literature.

### Motor learning practice

In the literature, many recommendations can be found on how physiotherapists should structure exercise sessions to optimise motor learning in stroke survivors (Levin & Demers 2021; Maier et al. 2019; Muratori et al. 2013; Winstein & Kay 2015). Most respondents followed recommendations from literature by decreasing the frequency of verbal feedback as a stroke survivor became more skilled in a movement (Sidaway et al. 2008) and by incorporating variability into exercise sessions with stroke survivors (Muratori et al. 2013).

However, most respondents did not follow recommendations from literature by not letting stroke survivors exercise at the recommended level of intensity or the recommended level of difficulty (KNGF 2014), not providing verbal feedback with an external focus of attention (Wulf 2013), not often incorporating task-specific training into exercise sessions (Dorsch et al. 2023; Scrivener et al. 2020) and not setting up the treatment environment to reflect the stroke survivor’s familiar home and/or community environment.

Overall, respondents inconsistently applied recommendations from the literature into stroke rehabilitation. This is consistent with earlier studies that reported a delay in the translation of knowledge gained via research and the implementation of that knowledge by physiotherapists working in neurorehabilitation (Fisher, Morton & Lang 2014; Levac et al. 2016). It was not in the scope of the study to investigate the reasons respondents followed or did not follow the recommendations from the literature regarding motor learning in stroke rehabilitation. However, a possible reason may be that physiotherapists have been unable to keep up with the large number of research published over the last 20 years detailing how to structure exercise sessions with stroke survivors according to motor learning principles (Kleim & Jones 2008; Kleynen et al. 2020; KNGF 2014; Levin & Demers 2021; Maier et al. 2019; Muratori et al. 2013; Winstein & Kay 2015). Practical considerations may also play a role (for example, high patient load or the amount of therapy medical aids are prepared to fund for stroke rehabilitation in the private health care sector may have made it difficult for respondents to achieve the recommended exercise intensity).

### Knowledge, attitude and practice of respondents who regularly treated stroke survivors

Most respondents who regularly treated stroke survivors had only moderate motor learning knowledge and inconsistently applied motor learning principles into stroke rehabilitation. Seven respondents are far too small a number to generalise these findings to the greater population of physiotherapists who treat stroke survivors on a regular basis. However, these findings may indicate a need for post-graduate training for physiotherapists treating stroke survivors to be able to confidently apply motor learning principles in stroke rehabilitation.

### Study limitations

The low response rate was a limitation of the study. Reasons for the low response rate may be physiotherapists’ high workload and limited time to complete surveys, as well as ‘survey fatigue’ among physiotherapists. Lack of availability of internet data at their workplace may be one reason, so few physiotherapists working in the public health care sector participated in the study. The low response rate may limit the generalisability of the findings and may not truly represent the knowledge, attitudes and practices of South African physiotherapists regarding motor learning. Another limitation of the study is that the survey was sent to all physiotherapists registered with the SASP. If the survey had been sent only to physiotherapists with a special interest in stroke rehabilitation, the outcome of the study likely would have been different.

### Recommendations

Respondents displayed a lack of knowledge in several important motor learning areas; post-graduate training is recommended for physiotherapists treating stroke survivors to be able to confidently apply motor learning principles in stroke rehabilitation. This post-graduate training may take the form of a continuous professional development activity for physiotherapists. Further research is needed to determine context specific implementation of motor learning principles in stroke rehabilitation.

## Conclusion

This study was the first study to describe the knowledge, attitudes and practices of South African physiotherapists regarding motor learning in stroke rehabilitation. Study findings revealed knowledge gaps in several motor learning areas. Further post-graduate training (for example, a continuous development activity for physiotherapists) is therefore recommended for physiotherapists treating stroke survivors to be able to confidently apply motor learning principles in stroke rehabilitation. Study results suggest that there is room for physiotherapists to apply motor learning principles more consistently into stroke rehabilitation. Future research should focus on exploring physiotherapists’ perceived barriers to the implementation of motor learning principles in stroke rehabilitation. This study may be a first step to developing strategies to enhance South African physiotherapists’ application of motor learning principles in stroke rehabilitation, which may ultimately lead to improve rehabilitation outcomes for South African stroke survivors.
